# SG-APSIC1151: ZeBox: A prophylactic device against airborne infection

**DOI:** 10.1017/ash.2023.106

**Published:** 2023-03-16

**Authors:** Srividya Janani Venkatraman, Arindam Ghatak, Debosmita Kundu, Santanu Datta

**Affiliations:** Biomoneta Research Pvt Ltd, Bangalore, India; Biomoneta Research Pvt Ltd, Bangalore, India; Biomoneta Research Pvt Ltd, Bangalore, India; Biomoneta Research Pvt Ltd, Bangalore, India

## Abstract

**Objectives:** Public health emergencies caused by airborne infectious agents are a significant concern, re-emphasized by the current COVID-19 pandemic. It is therefore vital to employ a technology that destroys microbes of all phyla and genera. We describe a novel technology called “Zebox” that can extract, trap, and destroy microbes from the air. This technology destroys even microbes that are resistant to known antibiotics. **Methods:** Airborne microbial load was enumerated using standard microbiological methods in both hospital ICUs and controlled conditions. Significant microbial reductions due to the ZeBox intervention in the ICUs were confirmed by statistical analysis. **Results:** ZeBox eliminated a broad spectrum of airborne pathogens (ie, viruses, bacteria, and fungi) in laboratory tests and in hospital ICUs, which are characterized by high, stochastic microbial loads. In closed-chamber experiments, ZeBox achieved a >99.999% reduction of airborne microbes. In the hospital ICU, ZeBox achieved a consistent >90% reduction across several months. Some of the airborne pathogens that ZeBox eliminated in the hospital ICU were multidrug resistant. **Conclusions:** ZeBox is an effective preventive technology against the spread of airborne pathogens and potentially associated infections. ZeBox could be used to reduce healthcare-associated infections in clinics and hospitals, as well as in burns units and immunocompromised patients. Zebox has the potential to be a significant prophylactic device in the global war on antimicrobial resistance.

